# Bayesian Spatial NBDA for Diffusion Data with Home-Base Coordinates

**DOI:** 10.1371/journal.pone.0130326

**Published:** 2015-07-02

**Authors:** Glenna F. Nightingale, Kevin N. Laland, William Hoppitt, Peter Nightingale

**Affiliations:** 1 School of Geography and Geosciences, University of St. Andrews, St. Andrews, Scotland, United Kingdom; 2 School of Biology, University of St. Andrews, St. Andrews, Scotland, United Kingdom; 3 School of Life Sciences, Anglia Ruskin University, Cambridge, England, United Kingdom; 4 School of Computer Science, University of St. Andrews, St. Andrews, Scotland, United Kingdom; Tianjin University, CHINA

## Abstract

Network-based diffusion analysis (NBDA) is a statistical method that allows the researcher to identify and quantify a social influence on the spread of behaviour through a population. Hitherto, NBDA analyses have not directly modelled spatial population structure. Here we present a spatial extension of NBDA, applicable to diffusion data where the spatial locations of individuals in the population, or of their home bases or nest sites, are available. The method is based on the estimation of inter-individual associations (for association matrix construction) from the mean inter-point distances as represented on a spatial point pattern of individuals, nests or home bases. We illustrate the method using a simulated dataset, and show how environmental covariates (such as that obtained from a satellite image, or from direct observations in the study area) can also be included in the analysis. The analysis is conducted in a Bayesian framework, which has the advantage that prior knowledge of the rate at which the individuals acquire a given task can be incorporated into the analysis. This method is especially valuable for studies for which detailed spatially structured data, but no other association data, is available. Technological advances are making the collection of such data in the wild more feasible: for example, bio-logging facilitates the collection of a wide range of variables from animal populations in the wild. We provide an R package, spatialnbda, which is hosted on the Comprehensive R Archive Network (CRAN). This package facilitates the construction of association matrices with the spatial x and y coordinates as the input arguments, and spatial NBDA analyses.

## Introduction

Social learning can be defined as learning that is facilitated by observation of, or interaction with, another individual, or its products ([1] modified from [2]). This can sometimes result in the spread of behavioural traits throughout groups, a process that we term ‘social transmission’. Recently, the focus of much social learning research has been the possibility of animal traditions and culture, with group-specific behaviour being found in a number of taxa, including primates (e.g. [3]), cetaceans (e.g. [4]) and birds (e.g. [5]). Such group-specific behaviour patterns often appear to be the result of different behavioural innovations spreading through groups by social transmission. However, these cases are often hotly debated, with critics claiming that alternative explanations, such as genetic differences between groups, might account for the observed pattern ([6]). Such debates have motivated the development of novel methods for inferring the social transmission of behaviour in freely interacting groups ([1, 7, 8]).

The term ‘diffusion’ refers to the observed spread of a behavioural trait through a group irrespective of the cause of the spread. A diffusion curve plots the cumulative number of individuals having acquired the trait as a function of time. Initially, researchers used the shape of the ‘diffusion curve’ to infer whether social transmission of a trait has occurred ([9]). Here researchers had suggested that if an acceleratory pattern is observed then social transmission could be inferred. The reasoning is that if social transmission is occurring, as the number of informed individuals increases, there are more individuals to learn from, so the per capita rate of acquisition increases. Unfortunately, there are several reasons why false negatives and false positives might arise using this approach ([10] [9, 11, 12]), leaving diffusion curve analysis an unreliable method for inferring social transmission.

A more promising approach, which allows not only the detection but also the quantification, of social transmission, is Network Based Diffusion Analysis (*NBDA*: [11, 13]). NBDA infers social transmission when the times, or order ([13]), of acquisition of the trait by individuals in animal groups follow(s) a social network. Similar models have been used in the social sciences ([1]). The reasoning here is that, other factors being equal, individuals that spend time together will have better opportunities to learn from each other than individuals that do not. This approach has been used successfully to infer social transmission in a number of species (e.g. humpback whales: [14]; songbirds, *Parus* spp.: [14]; and sticklebacks:[15][16]).

Unfortunately, it is not always possible to obtain data directly on the associations, or rates of interactions between individuals, to derive a social network for use in an NBDA. An alternative approach is to infer social transmission from the spread of behaviour in space, rather than its spread through a social network, on the assumption than individuals close in space are more likely to learn from one another than individuals more distant from each other. The traditional approach to inferring social transmission from the spatial pattern of diffusion has been to apply a wave of advance model. This comprises testing for a correlation between the time at which the novel behaviour is observed at a location and the distance from a specified point of origin. Here, we argue that a wave of advance approach has a number of weaknesses, and develop an alternative approach for analysing the spatial spread of behaviour that overcomes these concerns. Our approach is to adapt NBDA for the purpose: here the network connections between individuals represent the spatial proximity between them, in a way that reflects likely opportunities for learning from one another.

One obvious weakness of the wave of advance approach is that it requires the researcher to identify a point of origin, or multiple points of origin, for the novel behaviour. This may be intractable if behaviour is likely to have been acquired by asocial learning on more than a few occasions. For instance, in a population of 10000, if 95% of all individuals acquired the behaviour by social transmission, then we would need to identify 500 points of origin, making the wave of advance approach impractical, even though social transmission plays a key role in the diffusion. Conversely, it is not necessary to identify all points of origin for NBDA: the model allows for the possibility that any acquisition event could have occurred by asocial learning.

In addition, the wave of advance model does not fully allow for stochastic variability in the diffusion. If, in the early stages of the diffusion, the behaviour happens to advance further in one direction (say, North) than in another directions, then this will not be taken into account when assessing the evidence for social transmission provided by later events. Under such circumstances the researcher might expect individuals further away to the North to acquire the behaviour before closer individuals to the South, but the wave of advance model would not allow for this. In contrast, in NBDA the rate at which each naïve individual (i.e. those lacking the behaviour) acquires the behaviour at a given time is a function of the current state of all other individuals in the population, so such stochastic variability is taken into account.

Thirdly, the wave of advance model does not allow for individuals being distributed unevenly over space: we would expect behaviour to spread more rapidly over densely populated areas than over sparsely populated areas. Conversely, in the spatial NBDA that we develop, an individual’s rate of acquisition is a function of the number of neighbours possessing the behaviour, not just their distance from the individual in question, allowing this issue to be accounted for. In this paper we present an extension of NBDA designed to analyse the spatial spread of behaviour in a population in which individuals’ spatial locations, such as nest sites or home ranges, are known, but their patterns of interaction have not been directly measured. There is now an increase in the amount of data collected on animals in the wild due to the use of sophisticated data collection such as bio-logging. Increasingly, technological advances facilitate the collection of a variable-rich data on ecological data, including spatial behaviour and diffusion data. Consequently, we anticipate the data necessary to utilize the spatial NBDA is likely to be increasingly available over subsequent years. We illustrate the application of spatial NBDA to a simulated dataset comprising a spatial point pattern of the location of each individual in a population and the times at which each individual is first observed to display a novel behaviour. In this paper we compare this adaptation of NBDA to the wave of advance method. Finally, we illustrate how an environmental covariate can be incorporated in the spatial NBDA analysis. The incorporation of an environmental covariate would account for spatial environmental heterogeneity.

### 1.1. Spatial NBDA specification

NBDA typically seeks to quantify the effect of inter-individual social associations on the rate at which individuals within a given population display novel behaviour ([1]). In the spatial variant of NBDA that we present below, the inter-individual associations are estimated using the area of intersection between the zones of influence for each individual. In this scenario, two individuals are considered to be more able to exchange information when they reside in close spatial proximity than when they do not ([17, 18]). Each individual is assumed to possess a zone of influence ([19]), which is denoted by a disc of radius r (termed the ‘interaction radius’). The zone of influence of an individual can be thought of as the area within which this individual obtains nutrients, exerts its influence, or in this context, communicates information, and in functional terms it may relate to a territory or home range.

**Fig 1:**
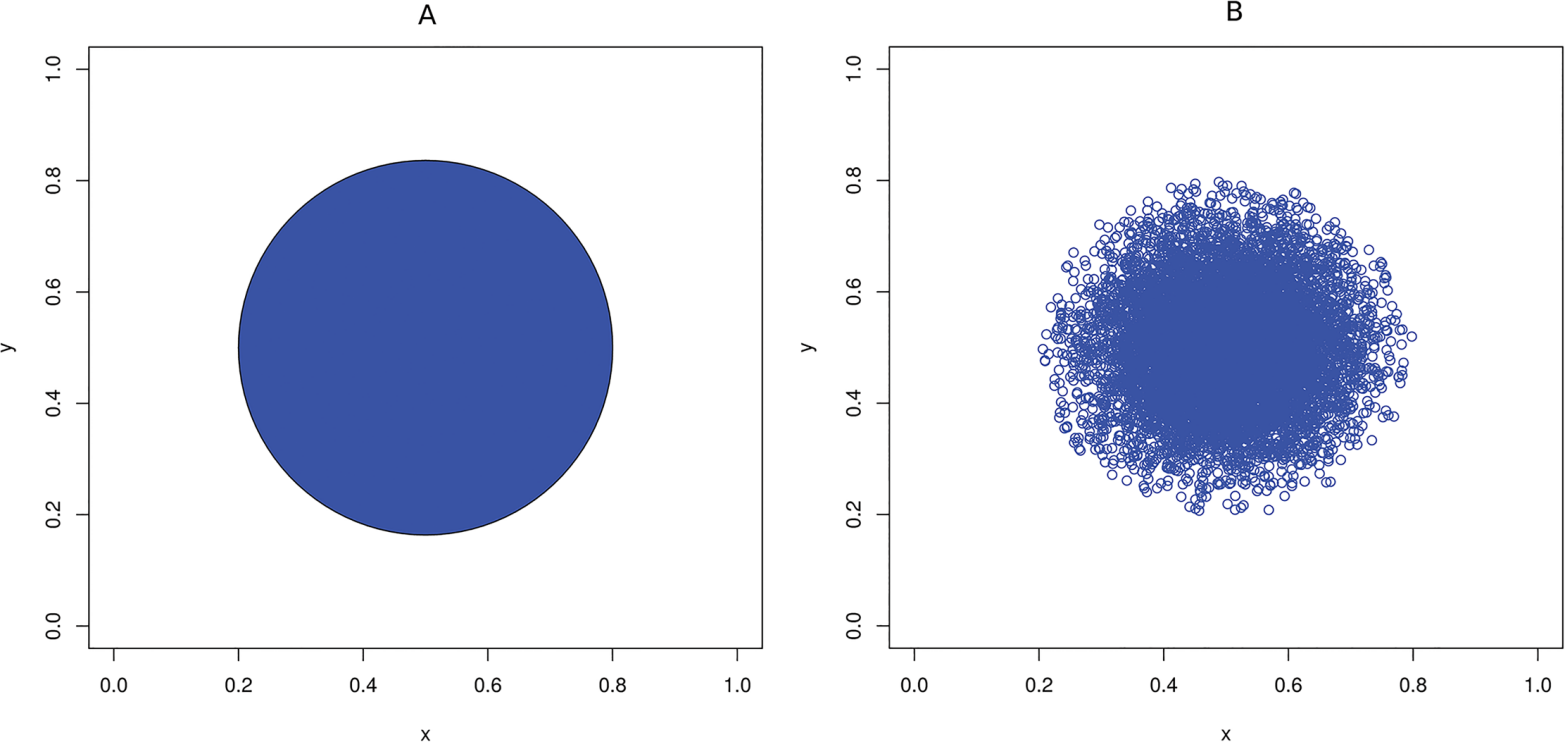
Zone of influence. (A) Zone of influence where the influence is constant within the entirety of the zone, and (B) Zone of influence where the influence decreases as the distance from the centre of the zone increases.

We consider one possibility, in which an individual’s influence is constant within the zone of influence. An alternative approach would be to assume that the influence can radiate at different intensities as the distance from the centre of the zone of influence changes. Fig 1 shows the difference in the two concepts where constant influence is denoted by a disc with points, too numerous to differentiate from each other.

**Fig 2:**
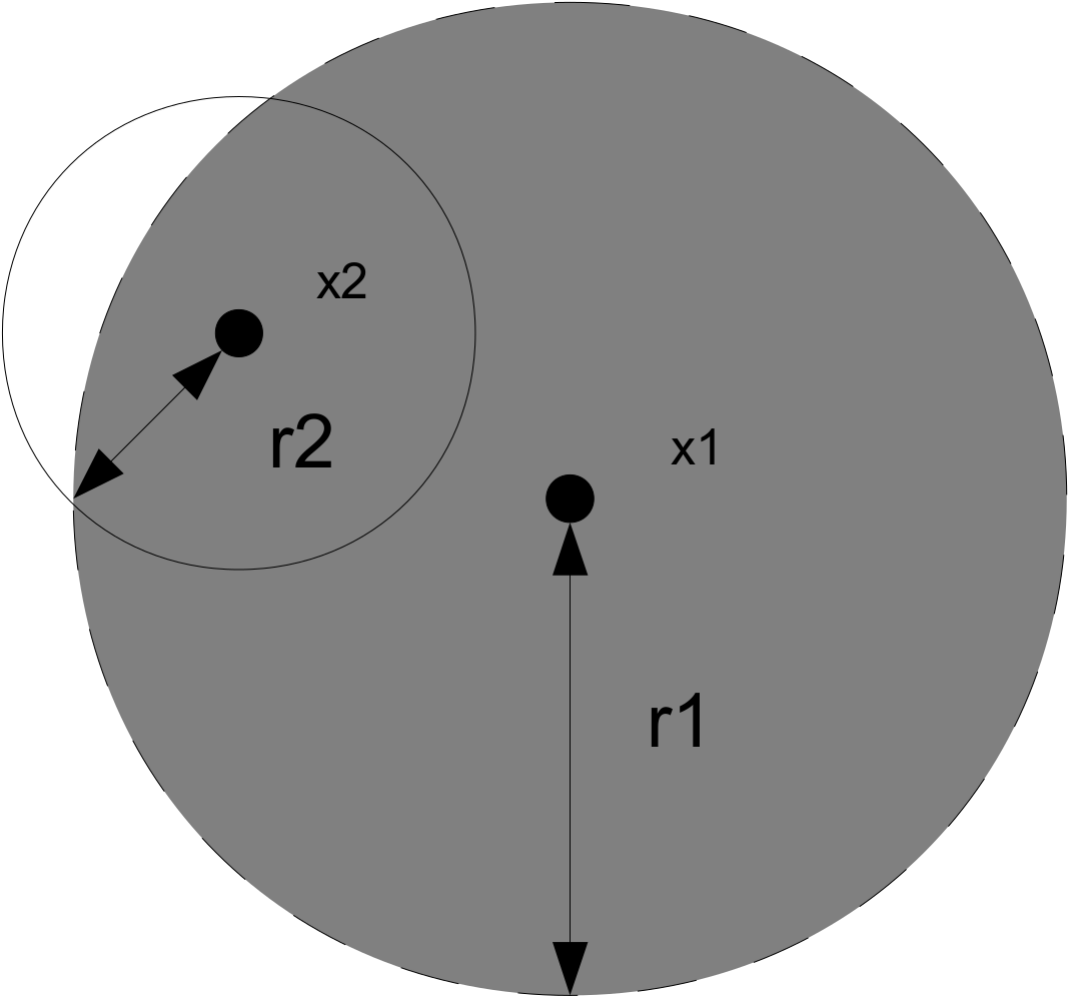
Asymmetric interaction between two individuals, x1 and x2. The interaction between individual x1 on x2 is calculated as the area of overlap between the two zones of influence and divided by the area of the zone of influence of individual x2. The proportion of area of overlap is greater for individual x2 than x1, as x2 has a smaller interaction radius.

Here we define the inter-individual association, a_ij_, of one individual, j, on another individual, i, as the normalised area of the intersection of the zones of influence of i and j divided by the total area of the zone of influence of individual j. The magnitude of this influence intuitively reflects the proportion of time i spends under the influence of j, and thus the rate at which novel behaviour is transmitted from j to i. The inter-individual influence can be symmetric or asymmetric ([20]). That is, the interaction radius for each individual may differ and as a result the proportion of the overlap between the zones of influence for both individuals would differ. The end result is that the effect of individual j on i need not be equal to that of i on j. Fig 2 illustrates this concept.

### 1.2. NBDA models

We conduct a NBDA based on the model specification and parameterisation described in [1]. (We extend the time of acquisition diffusion analysis (TADA) variant of NBDA here, which takes as data the exact times at which each individual acquires the trait, rather than just the order of acquisition). In this specification, the hazard function is a function of the baseline (asocial) rate of learning *λ*_0_, and s, the relative rate of the social transmission per unit of association of a given individual with other individuals that have already performed a desired behaviour. We define the hazard function as the instantaneous rate at which an individual solves or displays a novel task/behaviour, providing they have not done so before. In this paper we consider the baseline rate of learning to be a constant, *λ*_0_, however, this rate can be modelled as being non-constant. For an individual i at time t, and association matrix *A*, the hazard function *λ*_*i*_ (t) specifying the rate of performance of the behaviour is:

$$\lambda_{i}(t)=\lambda_{0}(1-z_{i}\;(t))(s\;\sum_{i\neq j}a_{ij}z_{j}(t)+1),$$

where *z*_*j*_(*t*) = 0 if j has not performed the desired behaviour and 1 if j has performed the desired behaviour, and *a*_1*ij*_ (t) provides the associations of individual i with each individual j which has already learnt the behaviour by time t.

As we use a Bayesian approach, we adopt a re-parameterization such that *s*′ = *λ*_0_ *s*:

$$\lambda_{i}(t)=(1-z_{i}\;(t))(s\prime \sum_{i\neq j}a_{ij}z_{j}(t)+\lambda_{0}),$$

where the term s’ is interpreted as the rate of social transmission per unit association with learned individuals within association matrix A. [21] introduce this re-parameterisation for a Bayesian NBDA to make setting of priors more intuitive. For this analysis we also incorporate an environmental covariate, *c*_*i*_, and denote its effect on the hazard function by the parameter *β*. The values of the environmental covariate were normalised. The hazard function then becomes:

$$\lambda_{i}(t)=(1-z_{i}\;(t))(s\prime \sum_{i\neq j}a_{ij}z_{j}(t)+\lambda_{0}\exp(\;\beta \;c_{i})).$$

For the calculation of the normalised area of overlap between each unique pair of individuals in the point pattern we utilize a Monte Carlo approach which is detailed in the documentation for the R package SocialNetworks. Other existing methods include Voronoi tessellations and polygon clipping algorithms ([22])

Since the analysis was conducted in a Bayesian framework, prior distributions for each parameter were set. The parameters, *λ*_0_, s’, and *β*, denoting the baseline rate, social rate and environmental effect respectively, were all assigned Uniform priors such that:

$$\log\;\beta ,\log\;s\prime ,\;\log\;\lambda_{0}∼U(-10,\;10).$$

An inherent feature of Bayesian analyses is the process of parameter updating. This is the method in which plausible values for the parameters are generated. This is aided by the appropriate specification of tuning parameters. In this analysis the tuning parameters varied for each parameter and for each model. For example, for model 3, the tuning parameter for the proposal distribution for the environmental parameter is 3. Since we used a Uniform random walk proposal update, the resulting proposal distribution is U[β-3, β+3]. In general we can describe the proposal distribution used for each parameter as U[α-ε, α+ε] where α denotes the parameter of interest and ε denotes the tuning parameter. The tuning parameters are chosen during an initial pilot tuning exercise so as to optimize the mixing of the Markov chains.

In this analysis a reversible jump MCMC algorithm was used to achieve model discrimination. Essentially, RJMCMC involves treating each model as a parameter (in addition to the already existing parameters). After the RJMCMC algorithm is run, the model which receives the highest support, that is, the model which has the highest probability is determined to be the best candidate model to describe the data. Terms like “Bayes’ factors” are also measures used to describe the comparison between two specific models.

Finally, in this analysis we remove the initial simulated values generated during the updating process (called burn-in) and use the remaining values to obtain posterior summaries of the parameter estimates which are presented in the Results Section of this paper.

### 1.3. Data for a spatial NBDA

For our spatial NBDA, the researcher requires data giving the central locations of individuals in a population, henceforth referred to as ‘nests’, and the times at which each individual displays the focal behaviour for the first time. The collection of the nest locations constitutes a point pattern of nest locations. To account for an observed environmental covariate, the researcher additionally requires the values of that variable at particular points in space. Ideally this would be measured at the nests, but if there is environmental collected within the area, but not at the specific nest locations, these values (values of the environmental covariates at nest locations)can be interpolated using, for example, a generalised additive model ([23]).

In order to enter these data into the spatial NBDA given above, we need to derive the values of a_ij_, which in turn requires us to choose the appropriate radius of interaction for each individual, so the normalised area of overlap can be calculated in each case. We suggest a method for determining an appropriate radius of interaction for each individual in Section 2. This method is used also when analysing point pattern using Markov point processes since the specification of an interaction radius is crucial to modelling Markov point processes.

## Exploratory analysis

An exploratory analysis was conducted so as to inform the choice of interaction radius specified for the construction of the social network used in the NBDA and to provide an indication of the nature of the inter-individual interactions amongst the individuals represented by the nests.

We obtain point pattern second order summary statistics on the observed pattern of nest locations by plotting the pair correlation function for the said point pattern. Point pattern second order summary statistics and the pair correlation function are discussed in detail below. Finally, we model the data using area interaction point processes to formally estimate the nature of the overall interactions of the individuals under study. In this model there is one population interaction parameter which describes whether the individuals are attracted to each other or are repelled from each other in general. This would corroborate the second order point summary statistics and provide insight into the NBDA results.

### 2.1. Point pattern second order summary statistics

**Fig 3:**
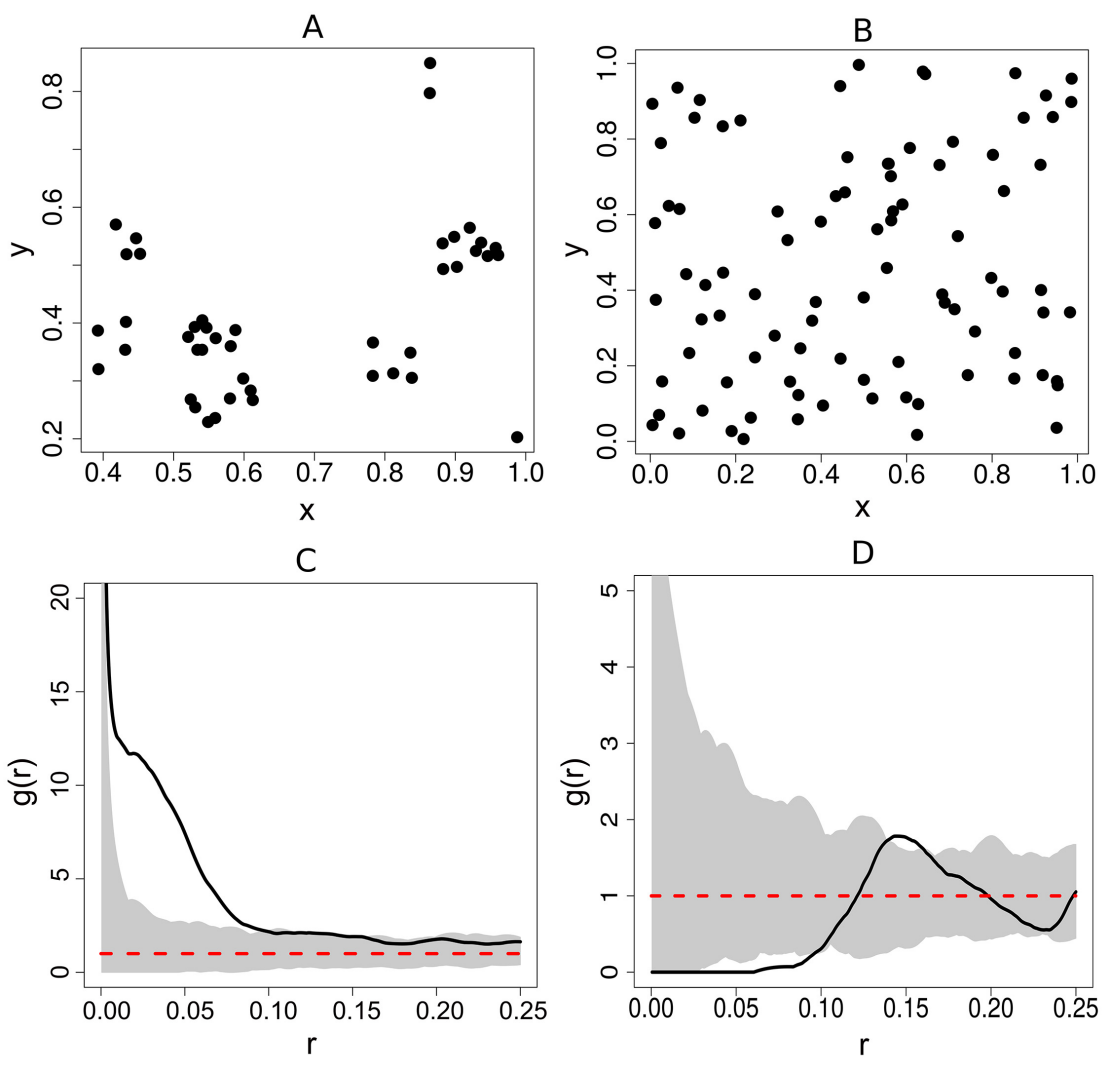
Plot of pair correlation functions for two different point patterns. The pair correlation function, denoted, g(r) is a function of an inter-point distance (r). The solid line represents the plot of the pair correlation for the data, whilst that for a point pattern generated from a homogenous Poisson point process is denoted by a dotted line. The two point patterns are depicted in (a) and (b) and the corresponding pair correlation plots are shown in (c) and (d).

Second order summary statistics for a set of points distributed in space are analogous to the concept of ‘spread’ or ‘dispersion’ in conventional statistics. That is, as with the concept of spread of data points in conventional statistics, the `spatial spread`of the points in space is captured by the point pattern second order summary statistics. These characterise the proximity of the points of the data to each other at varying spatial scales. This information is important to the spatial network based analysis (SNBDA) because it provides an indication of whether there is spatial dependence between the points. The structure of the spatially derived social network using the method of construction described in Section 1.1 would be influenced by the degree of spatial dependence of the corresponding nest locations. Most importantly, from the pair correlation function we can obtain an estimate of the interaction radius which best describes the dependence of the points in the pattern. The plot of the pair correlation for two different point patterns of nest locations is shown in Fig 3. This function takes values from zero to infinity. Values below 1 indicate spatial regularity (interaction of repulsion), values above 1 indicate clustering (interaction of attraction) and a value of 1 indicates complete spatial randomness.

For this analysis the second order summary statistic considered is the pair correlation function ([24]). The pair correlation function, g(r), can be expressed as: $g(r)=\frac{K\prime (r)}{2\pi r}\;\forall \;r\geq 0$

where K(r) is Ripley’s K function such that

$$K(r)=\xi^{-1}E[\#\;of\;points\;within\;distance\;r\;of\;an\;arbitrarily\;chosen\;point\;]$$

and *ξ* is the intensity of the point pattern or the expected number of points per unit area.

In contrast to the above description of K(r) for Ripley’s K function, the pair correlation, g(r) can be described as:

$$g(r)=\frac{intensity\;of\;points\;at\;distance\;r\;from\;randomly\;chosen\;point}{\xi }$$

The pair correlation function gives a summary of the spatial distribution of points in the point pattern at varying spatial scales. This summary is done for each point in the point pattern (of nest locations) and is commonly depicted as a plot. This is like plotting a ‘bird’s eye view at varying spatial scales’.

In a nutshell, for a given value of distance r, and for each focal point, the number of points at distance r from the said focal point is obtained. This is done at varying values of r (different spatial scales) and summed over all the points in the point pattern and divided by *ξ* to obtain the estimate of Ripley’s K function. This is then used to obtain g(r) as described earlier.

For each plot in Fig 3, the pair correlation function is plotted for the observed data (nest locations) indicated by a solid line and for a point pattern generated from a homogenous Poisson point process, indicated by a dotted line. The homogeneous Poisson point process is considered the null, or reference model in point process statistics which generates point patterns that display complete spatial randomness (CSR). Note that for each plot a simulation envelope which is constructed by plotting the pair correlation function for 1000 point patterns displaying CSR was included in each plot. If the empirical (observed data) pair correlation plot was above this envelope then there would be evidence of clustering in the point pattern at the value of r in question, if the plot fell below the envelope, then there would be evidence of repulsion. Finally, if the plot remains within the envelope, then there is no evidence of spatial structure in the point pattern and we conclude that the observed point pattern displays CSR.

The point pattern depicted in Fig 3(A) is a clustered one and from the plot of the pair correlation function, it is clear that this pattern is clustered between inter-point distances of 0.01 and 0.11 units and 0.13–0.15 units. This is because the plot lies above the Poisson reference line. This clustered effect seems most likely at a value of r = 0.05 units. A plausible interaction radius for this point pattern would therefore be r = 0.05 units.

In contrast, the point pattern depicted in Fig 3(B) has points which are regularly spaced and from the plot of the pair correlation function, it is clear that this pattern is ordered (due to repulsion between the corresponding individuals) at inter-point distances of 0.04–0.10. This is because the plot lies below the Poisson reference line. In this example, the choice of the interaction radius would be based on the fact that the empirical plot of the pair correlation function falls predominantly below the dotted reference line and in some cases below the simulation envelope. Plausible values for the interaction radius would be between 0.04 and 0.10 units. Since the emphasis is on local associations between individuals, large spatial scales should be avoided. This point pattern is often used as a canonical example of a regular or ordered point pattern and represents the locations of the centers of biological cells ([25]).

### 2.2. Area interaction point processes

Point process models are mathematical models that facilitate the formal analysis of points generated from the location of objects or events in space ([26]). A point process model is considered to be a random measure, such that each point pattern resulting from the model (i.e. each realisation of the point process) is different from each other. Despite this, each of the point patterns generated from a given point process model would exhibit similar structure in terms of intensity and dependence between points.

The simplest point process model is the homogenous Poisson point, where the probability a point will fall at a given location is constant across space and independent of the location of other points. This process is commonly used as a reference or null model in point pattern analysis. This concept is exemplified in the exploratory analysis in this paper when the pair correlation function is plotted for the data and for 1000 realisations of a homogenous Poisson model for the same number of points as that of the empirical dataset, allowing us to assess graphically whether the observed point pattern differs from the pattern expected if the pattern displayed complete spatial randomness (CSR).

Area interaction point process is used to estimate the interaction or dependence between the individuals represented by the spatial point pattern generated by the data. Area interaction point processes are point process models that model not only the intensity of the points on a given spatial point pattern, but the dependence or interaction between the objects or events which are represented by those points.

In an exploratory analysis we fit the spatial point pattern of the nests considered to a univariate area interaction point process model using the R package *spatstat*. The exploratory analysis provides an indication of the structure of the spatial point pattern generated by the data. For example, the point pattern may be spatially clustered, regular or random. The estimate of the interaction parameter generated from an area interaction point process would provide an indication of the overall interaction between the individuals represented by the points. That is, is their overall interaction one of repulsion or attraction? Or is the spatial structure indicative of complete spatial randomness (CSR) as exhibited from point patterns generated from a homogenous Poisson point process model. Note that the assumption of this class of point processes is that the spatial distribution is a function of the inter-individual interaction and other covariates if appropriate.

Area interaction point processes ([22, 27, 28]) have been described as being appropriate for modelling biological processes. For example, a recent study of nest locations of gorillas ([29]) employed this class of point processes to estimate the overall inherent inter-individual interaction.

The univariate area interaction point process facilitates the estimation of the intensity and the overall inter-individual interaction from the observed data. For this analysis we also incorporate an environmental covariate c with corresponding parameter, *β*. The likelihood for the area interaction point process is defined as:

$$f(\nu )\propto \xi^{n(\nu )}\gamma^{-|U_{\nu ,r}|}\beta^{c}$$

where *ξ* and *γ* denote the intensity and interaction parameters, and *n*(***ν***) denotes the number of points in the point pattern. The term |*U*_*x*,*r*_| is expressed as

$$|U_{\nu ,r}|=\overset{n}{\underset{i=1}{⋃}}B(\nu_{i},r),$$

where *B*(***ν***_*i*_, *r*) is a disc or radius r centered at each data point on the point pattern ***ν***. This can be thought of as the area of the union of discs of interaction radius r (determined for example graphically with the plot of the pair correlation function) centered at each data point ([28]). This leads then to the expression for the area of a disc centered at radius r:

$$B(\nu_{i},r)=\{a\in R^{2}\;:\;|\left|a-\nu_{i}\right||\leq r\}.$$

## Application of Spatial NBDA to Simulated Data

### 3.1. Data

We use three datasets, dataset_1, dataset_2, and dataset_3 to illustrate the spatial NBDA method. We conduct the full analysis on dataset_1, and illustrate specific issues with the remaining datasets. We demonstrate the effect of analysing a dataset where there is no inherent inter-individual associations in dataset_2, and that of using multiple values of the interaction radius, r.

The data for dataset_1 consist of a spatial point pattern representing the nest locations of individuals in a population, values of an observed environmental covariate at particular points in space, and the times at which each individual displays the focal behaviour for the first time. The bivariate function, E(x,y), of the spatial coordinates x and y, gives the value of the environmental covariate at the location of coordinates c(x,y). For the environmental covariate in this analysis, E(x,y) is defined as:

E(x,y)=3x-2y.

**Fig 4:**
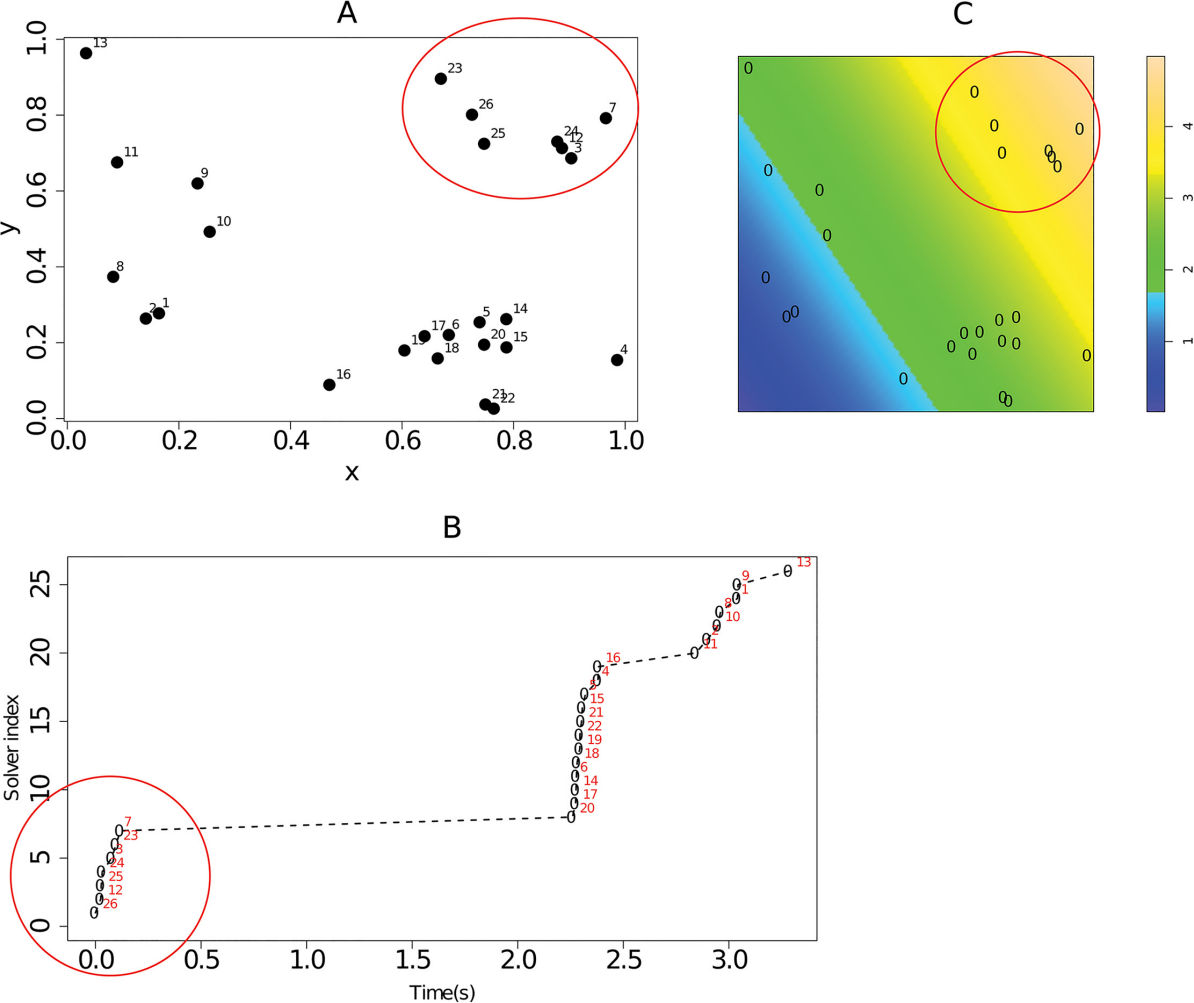
Dataset_1. (A) Spatial point pattern of nest locations. Each point represents the location of the home base of one of the 26 individuals considered. The number associated with each point on the plot represents the unique id of the individual at that nest. (B) diffusion times for each individual for diffusion 2. (C) simulated image of an environmental covariate (with point pattern superimposed). The image indicates that there is a gradient in the values of the covariate from left to right (diagonally).

The point pattern consists of 26 points, with each point representing an individual's nest location, associated with a disc of radius r (interaction radius). The inter-individual associations are calculated using the area of the disc per individual and the overlap of the discs for a given pair of individuals. The spatial point pattern, environmental covariate map, and plot of diffusion times (one of the ten diffusions considered) are shown in Fig 4. From this figure it is clear that the times at which individuals acquired the studied behaviour occurred in spurts which correspond to the spatial clusters of nest/home base locations. Consider for example, the times for diffusion 2, shown in Fig 4(B). The first cluster of times is circled in red on the plot. These correspond to the cluster of nest locations in the upper right hand corner of Fig 4(A), also circled in red. In addition, it is seen that there are more nest sites in the areas with comparatively lower environmental covariate values as shown in Fig 4(C). These observations motivate the use of a modelling approach which extends the current implementation of NBDA model and take into account for the spatial distribution of nest locations and also possible additional environmental covariate information.

The data were constructed by:

- Simulating a clustered point pattern using a Matern-cluster point process (to represent locations of nests for example),
- Constructing a spatially derived social network using the xy coordinates on the point pattern,
- Simulation of event times for multiple diffusions using the spatially derived social network. Gillespie’s algorithm was used for this.

Dataset_2 and dataset_3 are constructed in a similar fashion to dataset_1. Key differences are due to the fact that dataset_2 was constructed using a Poisson point pattern and no inherent inter-individual associations, and dataset_3 was constructed using a uniform environmental covariate values.

### 3.2. Methodology

#### 3.2.1. Dataset_1

To analyse these data first we conduct an exploratory analysis to obtain a preliminary indication of the spatial dependence of the points in the observed pattern of nest locations. To achieve this we consider a second order summary statistic for point patterns, using a pair correlation function ([24, 26]). This is an important component of the analysis since the plot of the pair correlation provides a description of how clustered or regular the pattern is at different spatial scales. The choice of the interaction radius used in constructing the social network would be obtained by selecting the value of r that best describes the observed point pattern.

As discussed earlier, the plot of the pair correlation function gives an indication of the dependence between the points in the pattern. This is used routinely in conjunction with biological/ecological background information where available ([27]). Recall that each point represents a given individual (i.e. its nest location). For this analysis the plot of the pair correlation function will be compared with that of a dataset (spatial point pattern) that displays complete spatial randomness (CSR). Such a point pattern is simulated from a Poisson point process, which is typically considered a ‘reference/null model’ in point process statistics.

We then conduct an NBDA where we consider three models, with (1) baseline learning rate alone, (2) baseline rate plus social transmission, and (3) baseline rate, social transmission and environmental covariate, as parameters in the model. That is, model 1 (the null model) contains the parameter set ***θ*** ^**1**^ = {*λ*_0_}, model 2 which contains the parameter set ***θ*** ^**2**^ = {*s*′,*λ*_0_} and model 3 (the full model) contains the parameter set ***θ*** ^**3**^ = {*s*′,*λ*_0_,*β*} (See Equation 3). Finally, we use an RJMCMC algorithm to achieve model discrimination.

#### 3.2.2. Dataset_2, dataset_3

For dataset_2 we consider two models:—with identical parameterization as models 1 and 2 in that for dataset_1. The aim is to demonstrate the effect of estimating a social effect in a dataset that does not inherently contain inter-individual associations. For dataset_3, we consider the same two models, but conduct a sensitivity test for the interaction radius r, and demonstrate the effect of changing the scale (value of r) on the estimation of parameters of interest.

## Results – dataset_1

### 4.1. Exploratory analysis

We now present the results for the exploratory analysis in the following sub sections.

#### 4.1.1. Pair correlation function

**Fig 5:**
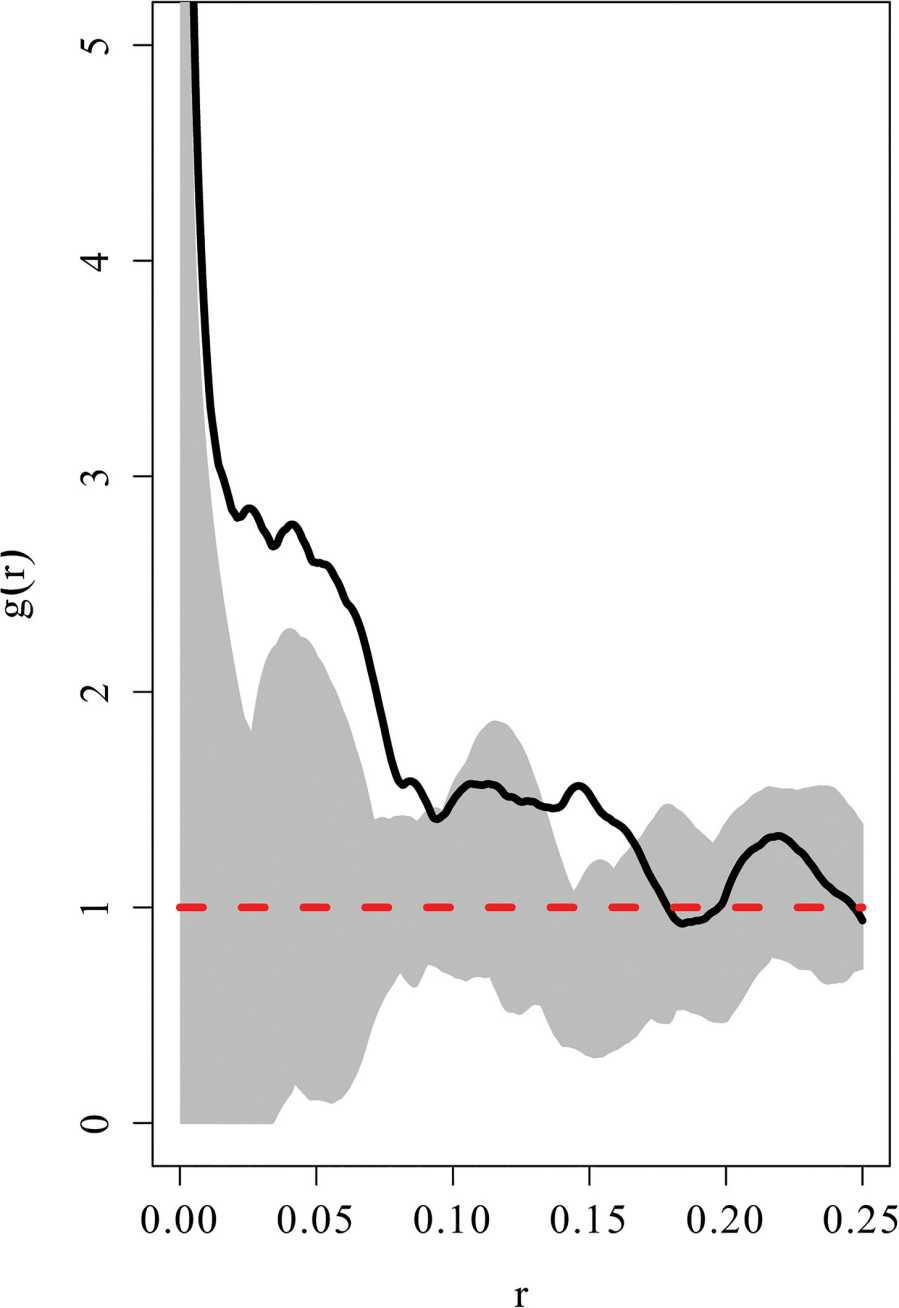
Plot of the pair correlation function for the data including a Poisson reference line (dotted) and a simulation envelope of 100 realisations of a point pattern displaying CSR. The empirical pair correlation is indicated by the solid line.

Fig 5 shows the plot of the empirical pair correlation function for the data. A simulation envelope (100 simulations) from a Poisson model is also included.

The plot for the data (solid line) is above the Poisson reference line and above the simulation envelope for distances between 0.02 and 0.09 units, and also for distances between 0.14 and 0.16. In particular for these distances the spatial pattern is more clustered than expected for a randomly distributed set of the same number of points within a similar area. As a result of this we choose an interaction radius of r = 0.05 to calculate the inter-individual associations in the social network used for the NBDA.

From this analysis we have some evidence of the behaviour of the point pattern at various spatial scales. The observed clustering in the point pattern suggests that there is some degree of attraction between the individuals concerned. We now fit this point pattern to an area interaction point process model so as to estimate the effect of underlying inter-individual interactions and an environmental covariate on the spatial distribution of the observed points. We’re interested in the question: What is the quantified effect of the inter-individual interactions and the observed environmental covariate on the spatial distribution of the observed nest locations? This is important for the specification of the function used to estimate the individual associations attributed to each unique pair of individuals to construct a social network for the NBDA. If the environmental covariate is important, then the interaction function should have an environmental component incorporated.

#### 4.1.2. Area interaction point process

When the spatial point pattern observed was fitted to an area interaction point process (r = 0.05 units), the log of the estimate of the interaction parameter (*γ*) was found to be greater than 1. The log estimate accompanied by 95% credible intervals is 3.361(1.632,5.089). In this instance the interaction parameter is a quantification of the overall interaction between individuals represented by the points in the point pattern. This interaction can be one of attraction (due to facilitation for example), repulsion (due to competition, for example) and negligible. In general, positive values indicate that there is an interaction of attraction (facilitating clustering of individuals) whilst negative values signify an inter-individual association of inhibition (facilitating spatial regularity). The choice of the interaction radius for this analysis and for constructing the social network was based on the plot of the pair correlation function where the pattern was observed to be clustered at 0.05 units. Based on the estimate of the log of the interaction parameter, we can say that the overall inter-individual interaction is one of attraction. The other parameter in this model which accounted for any impact of the environmental covariate (*β*) on the spatial distribution of the points was found to have a log estimate (and 95% credible intervals) of –0.231(-1.406,0.944). This negative value suggests that the environmental covariate has a negative effect on the density of points observed.

From this analysis we note that the overall inter-individual interaction within the population under consideration is of attraction. In addition, the estimate of the environmental covariate suggests that at higher values of the covariate, there are fewer nests. These facts justify our use of a spatially derived social network for the NBDA. The use of a spatially derived social network would take into consideration the associations amongst the individuals and the incorporation of an environmental covariate would account for any effects which are due to the environmental heterogeneity with respect to that covariate.

### 4.2. Bayesian NBDA analysis

Recall that three models were considered. Model discrimination was achieved through the use of an RJMCMC algorithm (with 20,000 simulations). Two of the three models considered received posterior support. These are model 2 (posterior probability of 11172/18001), and model 3 (posterior probability of 6829/18001). Clearly, the model that received the highest posterior support was model 2. Model 1, the null model, which contains only the baseline rate parameter, received no posterior support. Recall that Model 2 contains the baseline rate and social effect parameter while model 3 contains the baseline rate, social effect and environmental effect parameters.

**Fig 6:**
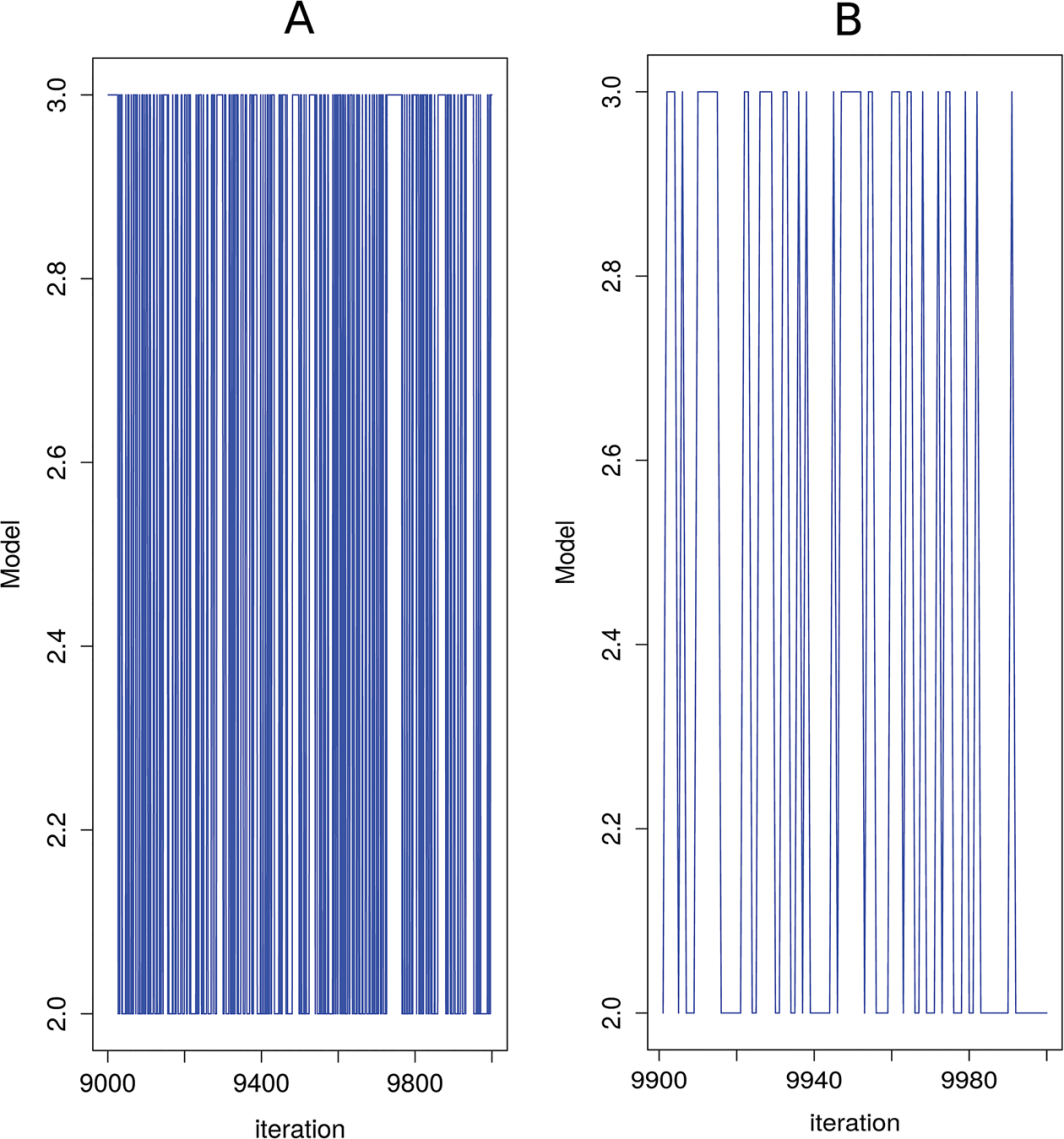
Model trace plots. (A) Model trace plot (for the last 1000 iterations of the simulation) for model discrimination between models 2 and 3. (B) Model trace plot (for the last 100 iterations of the simulation) for model discrimination between models 2 and 3.

Finally, the Bayes factor in favour of model 2 against model 3 is 1.64 which suggests that the strength of the posterior evidence in favour or model 2 against model 3 is minimal. Fig 6 shows the model trace plot for the model discrimination analysis for the last 1000 of the 20000 iterations employed.

The posterior parameter estimates for the three models are shown in Table 1.

**Table 1:** Mean posterior parameter estimates (in natural logarithm format) for the models considered.

Parameter	Model 1	Model 2	Model 3
Baseline rate	0.104(-0.023,0.225)	-1.599(-1.961,-1.262)	-1.972(-2.690,-1.401)
Social effect		0.839(0.692,0.981)	0.854(0.709,0.998)
Environmental effect			-2.853(-9.363,-0.678)

The 95% symmetric credible intervals are also included for each parameter.

### 4.3. Wave of advance analysis

A linear regression model was used to perform the wave of advance analysis. The independent variable is the time (for each individual) taken from the instance at which the innovator displayed the novel behaviour. The dependent variable is the distance (for each individual) from the spatial location of the innovator. The estimate of the coefficient representing the effect of the distance from the innovator is -0.631 with a standard error of 0.259(p value = 0.02). The coefficient of determination, R^2^ was 0.02 suggesting that the model has a poor fit.

## Results – dataset_2, dataset_3

**Fig 7:**
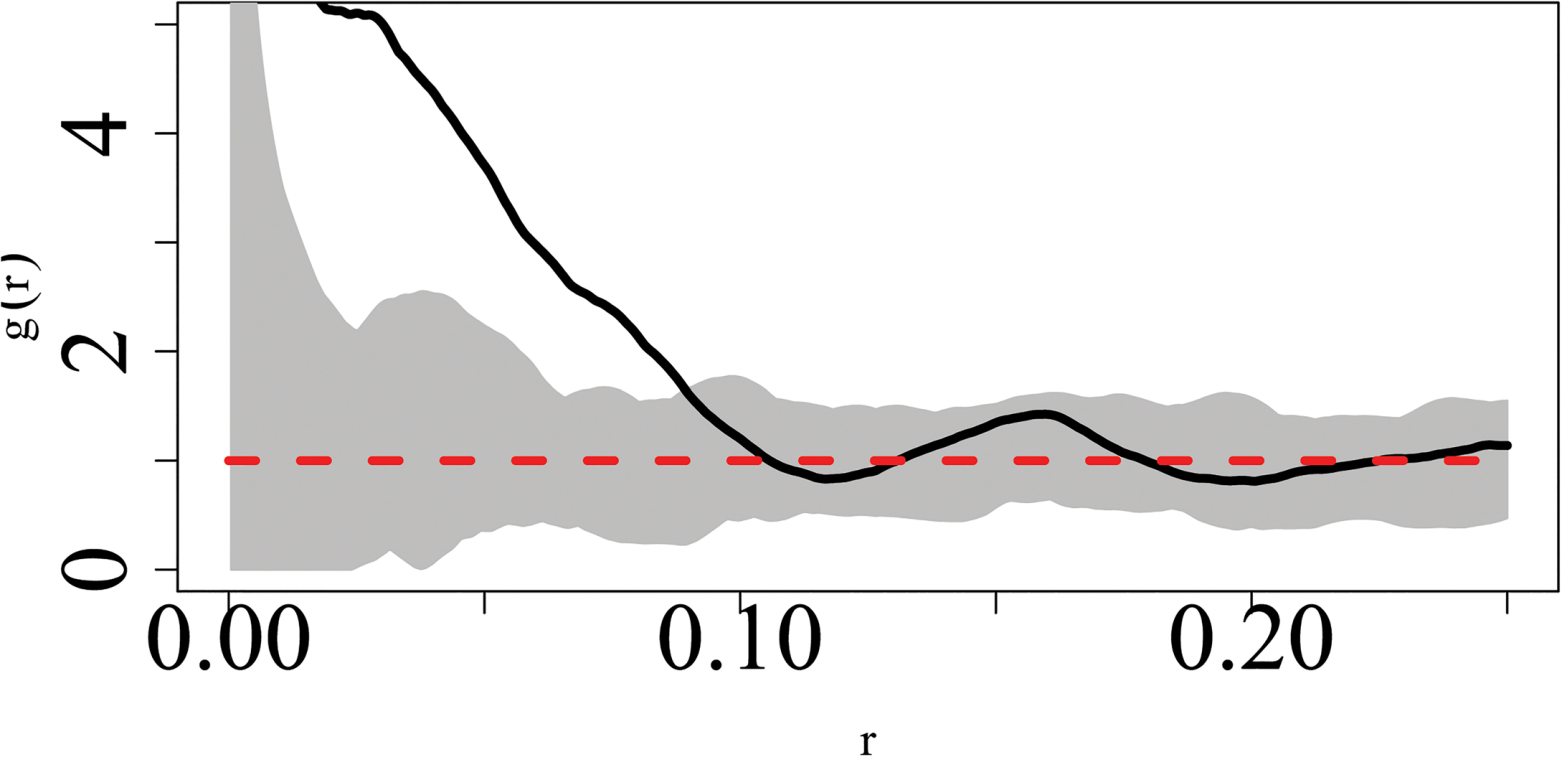
Pair correlation plot for dataset_3.

The posterior estimates for the analysis of dataset_2 and dataset_3 are shown in Tables 2 and 3 respectively. Fig 7 shows the pair correlation plot for dataset_3.

**Table 2:** Mean posterior parameter estimates (in natural logarithm format) for the models considered.

Parameter	Model 1	Model 2
Baseline rate	-9.188(-9.605,-8.845)	-9.209(-9.599,-8.864)
Social effect		0.09(9.9335,9.567)

The 95% symmetric credible intervals are also included for each parameter.

**Table 3:** Mean posterior parameter estimates (in natural logarithm format) for the models considered.

Parameter	Model 2(r = 0.02 units)	Model 2(r = 0.05 units)	Model 2 (r = 0.08 units)
Baseline rate	4.207(3.922,4.513)	3.712(3.366,4.152)	3.774(3.299,4.145)
Social effect	-1.805(-9.559,4.494)	6.031(5.182,6.706)	4.828(4.189,5.384)

The 95% symmetric credible intervals are also included for each parameter.

The results for dataset_2 in Table 1 show that there is a great amount of uncertainty in estimating the social effect, in particular, the corresponding symmetric credible interval reflects that of the prior. This clearly suggests that the ‘prior has been returned’ and there is no information in the data to suggest that there is a social effect.

The results for dataset_3 indicate that at the scale of r = 0.02, the social effect is very small and the symmetric credible is very large indicating that there is considerable uncertainty associated with this estimate. Referring to the pair correlation plot in Fig 7 we note that at this scale, the values of the pair correlation plot are not reliable with values of r > = 0.015 being undefined. For r = 0.05 and r = 0.08 we note that the posterior estimate of the social effect is positive, with that for r = 0.08 being lower than the former. This is supported in the corresponding pair correlation plot where the degree of clustering is highest at r = 0.05

## Discussion

### 6.1. Dataset_1

Overall, the results from this extension to NBDA illustrate the effect of spatially clustered individuals on the spread of a novel trait.

From the results we note that models 2 and 3 received the highest level of posterior support. This suggests that the social, and asocial parameters are important in describing the rate at which the trait is performed in the simulated population. Of course, we selected the dataset in this instance, but it serves to illustrate the methodology. The method is specifically applicable to territorial species for which a home base can be clearly identified. The method can handle cases in which the home base changes over time. In such cases, the calculation of the inter-individual associations would be adjusted to account for temporal effects.

The NBDA analysis shows that there is a measurable effect of the estimated social network on the rate at which the desired trait was displayed throughout the simulated population of individuals. This is corroborated by the results of the exploratory analysis. In particular, the second order point pattern summary statistics and the area interaction point process model results indicate that the overall interaction between the individuals represented by the point pattern is one of attraction. This knowledge potentially aids interpretation of the phenomena at hand. For instance, it might imply that the animals concerned were attracted to each other because of the potential information that could be gained from them. Alternatively, the animals might be attracted to each other for independent reasons (e.g. reduction of predation risk) that only incidentally facilitate information flow.

In our example, the model with the environmental covariate (model 3) also received posterior support. The environmental covariate had a comparably lower effect (compared to the social effect) on the rate at which the individuals solved the task at hand. For a unit increase in the value of the environmental covariate the rate at which the task was solved was increased by exp (-2.853). Obviously this need not be the case with a real dataset, and the method potentially allows researchers to quantify the impact of environmental variables on the acquisition of a trait.

In comparison, the wave of advance analysis, which computed the regression of time from origin against the distance from the innovator, misleadingly suggested that the distance from the innovator was not important in describing the time from origin. The failure of this method reflects the fact that additional processes needed to be considered, such as the possibility of both social and asocial learning processes were at work. In addition, in the wave of advance model, only one exemplar is considered: the innovator. A more realistic approach would be to consider the associations from all exemplars at each time point. Another advantage of NBDA is that this is done.

### 6.2. Dataset_2, Dataset_3

The results from the analyses of these two datasets confirm that the proposed method would reflect a situation where there is no inherent social effect, and to demonstrate multi-scale spatial NBDA. For the sensitivity analysis conducted for dataset_3, we see that the posterior estimates of the parameters of interest were dependent on the choice of r. This highlights the benefit of the use of the pair-correlation function in the analysis since it would provide information on the characteristics of the pattern at different scales. For example, at very small distances (small values of r) the pair correlation plot may be undefined This is an indication that these these distances are not ideal choices for r. The pair correlation plot may indicate that the pattern is clustered at one scale and regular (no clustering) at another. The use of different values of r in the NBDA would provide insights into the social interaction at various spatial scales.

### 6.3. Extensions

Possible extensions to this approach would include accounting for different groupings within the population (e.g. adults and juveniles, males and females, or sub-communities) by specifying different interaction radii for each group. The point pattern resulting from this type of categorization would be a ‘marked’ point pattern, where the ‘marks’ are at the group level and are categorical. For two groups we have a bivariate point pattern, for more groups we have a multivariate point pattern. Another possible extension to this approach is the inclusion of random effects. This could be done at the individual, group or temporal level and would have the advantage that factors such as individual learning aptitudes could be taken into account.
